# Changes in the soil bacterial community along a pedogenic gradient

**DOI:** 10.1038/s41598-017-15133-x

**Published:** 2017-11-06

**Authors:** Manuel Sánchez-Marañón, Isabel Miralles, José F. Aguirre-Garrido, Manuel Anguita-Maeso, Vicenta Millán, Raul Ortega, José A. García-Salcedo, Francisco Martínez-Abarca, Miguel Soriano

**Affiliations:** 1 Department of Soil Science and Agricultural Chemistry, University of Granada, E-18071 Granada, Spain; 2 Center for Intensive Mediterranean Agrosystems and Agri-food Biotechnology (CIAMBITAL), University of Almeria, E-04001 Almería, Spain; 3 CBS Universidad Autónoma Metropolitana-Lerma, Hidalgo Pte. 46, Col. La Estación, 52006 Lerma, Estado de México Mexico; 4 Pfizer-University of Granada-Junta de Andalucía Centre for Genomics and Oncological Research (GENYO), E-18016 Granada, Spain; 5 Molecular Ecology Group, Department of Soil Microbiology and Symbiotic Systems, Estación Experimental del Zaidín, Spanish Council for Scientific Research (EEZ-CSIC), E-18008 Granada, Spain; 6 Infectious diseases and Microbiology Unit, Biosanitary Research Institute ibs.GRANADA, University Hospitals of Granada/University of Granada, E-18012 Granada, Spain

## Abstract

Current research on the influence of environmental and physicochemical factors in shaping the soil bacterial structure has seldom been approached from a pedological perspective. We studied the bacterial communities of eight soils selected along a pedogenic gradient at the local scale in a Mediterranean calcareous mountain (Sierra de María, SE Spain). The results showed that the relative abundance of Acidobacteria, Canditate division WPS-1, and Armatimonadetes decreased whereas that of Actinobacteria, Bacteroidetes, and Proteobacteria increased from the less-developed soils (Leptosol) to more-developed soils (Luvisol). This bacterial distribution pattern was also positively correlated with soil-quality parameters such as organic C, water-stable aggregates, porosity, moisture, and acidity. In addition, at a lower taxonomic level, the abundance of Acidobacteria Gp4, Armatimonadetes_gp4, *Solirubrobacter*, *Microvirga*, *Terrimonas*, and *Nocardioides* paralleled soil development and quality. Therefore, our work indicates that the composition of bacterial populations changes with pedogenesis, which could be considered a factor influencing the communities according to the environmental and physicochemical conditions during the soil formation.

## Introduction

Soil bacteria, involved in the decomposition of organic residues, nutrient cycling, aggregation, and formation of humic substances, can reach up to 10 billion cells per gram^1–3^. However, little was known in the past about the composition and diversity of bacterial communities until culture-independent community-profiling approaches became available^4–6^. While only 1% to 10% of the bacteria present in a soil sample can be readily cultured under laboratory conditions, metagenomic methods are potentially suitable to distinguish all the species of a natural microbial community by the detection of specific DNA sequences^7^. The analysis of ribosomal RNA genes (rRNA), by using the latest advances in next-generation sequencing (NGS) technology, has shown that soil bacterial diversity in a natural setting is extremely high^8–12^, but the mechanisms controlling such diversity are poorly understood^13^.

Although a plethora of research has documented the influence of soil on the bacterial genotypic diversity, most of these studies have been concerned with single characteristics such as pH^14–16^, texture^17^, or pollutants^18–20^, whereas the joint influence of factors that are lumped in a soil survey^21^ remains unclear. Most soil microbiology studies have hardly reported pedological information about profile, typology, or genesis of soils from which the samples are taken^22^. However, laboratory tests have shown that the distribution of microbial taxa could be locally adapted to soil^23^. Likewise, several field studies have recently found significant differences in bacterial diversity between agricultural soils located in northern and southern China^9^, among six forest soils of North and Central America ranging from Colorado to Panama^13^, and at 12 sampling points between 1500 and 2600 m a.s.l. in the Pyrenees range^24^. Although these studies have sought to demonstrate the effect of historical contingencies and climatic gradients, examining the different soil-formation environments within each area according to its latitudinal and elevational variation, the inherent difference between soils may also have contributed to the genetic divergence of bacterial assemblages. Indeed, in cases when the effects of land use on distribution patterns of microbial communities have been evaluated^12,25,26^, the researchers have compared areas within the same soil typology. Given the complex interrelationships among soil microbes and site conditions, it was found to be unlikely that their variation stemmed from a single influence or a small set of factors. Everything suggests that bacteria are adapted to the soil they inhabit.

Accordingly, an improved understanding of bacterial relationships with the soil, considering the latter as an entity resulting from the interaction of environmental factors (parent material, vegetation, topography, and climate) over time, may help solve some outstanding challenges in soil microbiology. Such a pedological approach could be useful in studies aimed at establishing unifying principles in soil microbial ecology^27^, to determine the response of microbial communities to long-term environmental change^28^, and to clarify the relative significance of chance, environmental factors, and past legacies on prokaryotic biogeography and diversification^29^. However, the metagenomic bacterial exploration linked to a pedological study is still uncommon and the role of pedogenesis in shaping the bacterial community structure remains unknown.

To address this issue, we undertook a local-scale survey of soils which are widespread and have ecological significance within a Mediterranean mountain area on limestone. Here, topography controls microclimate, vegetation, water redistribution, and surface stability, which in turn leads to the formation of soils with different development. We searched for soils representing a pedogenic gradient in the Mediterranean region. To minimize the factors of biological variation due to distance, we chose soils with different genesis and development but also as close together as possible. Our study was aimed at analysing the profile, typology, and physicochemical parameters of each soil, as well as bacterial genotypic diversity in order to help elucidate the influence of pedogenesis on the bacterial assemblages.

## Results

### Soil development and quality

**Figure 1:**
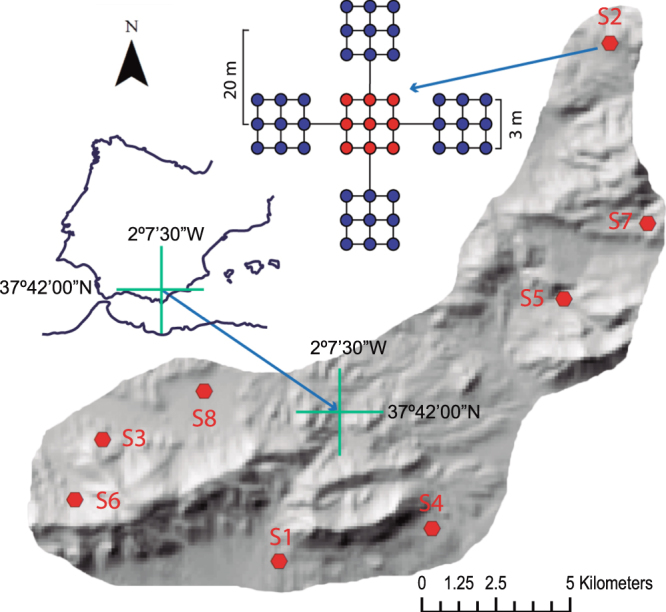
Sampling strategy and location. At each site (S1 to S8), we surveyed a soil profile (red points) and four composite topsoil samples were taken at 20 m from the soil profile (blue points). Each topsoil sample was composited from nine samples of the top 20 cm of soil collected in a plot of 3 × 3 m. Maps were created using ArcGIS v.10.2 software (http://www.esri.com/arcgis/about-arcgis).

We studied eight soils on limestone but each with different vegetation, altitude, slope, and topographic orientation (Supplementary Table S1) in the Sierra de María Natural Park (SE Spain, Fig. 1). The calcimorphic features of the accumulation of secondary carbonate such as filaments, nodules, and coatings were common in the profiles. The soil pH ranged between slightly and moderately alkaline (7.5–8.5) and the contents of equivalent CaCO_3_ usually increased towards the profile bottom (Table 1). The two least carbonate soils (S7 and S8) had horizon sequences Ah-Bt-R and Ap-Btk-Ck, reddish colours in accord with the amount of dithionite extractable iron (Fe_d_), a huge amount of clay, and strong angular-blocky aggregates. The Harden’s profile development index, which quantifies the degree to which the soil-horizon properties differ from the parent-material properties (C or R layer), was 25.2 for S7 and 25.7 for S8, both soils, respectively, classified as Leptic Luvisol and Luvic Calcisol^21^. They are broadly called red Mediterranean soils including a typical terra rossa on hard limestone (S7) and a red soil developed on Quaternary colluvial glacis later degraded by tillage (S8). Because their pedogenic processes including leaching of carbonate minerals, rubefaction, and clay accumulation require a long time period, they must be the oldest soils in the Sierra de María area. In other words, they had the greatest pedogenic change with respect to the soil parent material.

**Table 1:** Modal soil profiles of the pedogenic gradient studied in Sierra de María (SE Spain).

Soil	Depth (cm)	Munsell color (dry)	Structure^a^	Sand (%)	Clay (%)	Fe_d_^b^ (g kg^−1^)	Water retained		pH (H_2_O)	CO_3_^−2^ (%)	Org. C (g kg^−1^)	CEC (cmol_+_ kg^−1^)
							−33 kPa	−1500 kPa				
**S1 (PDI** ^**c**^ = **4.9)**												
Ah	0 − 15	9.8YR 3.8/2.5	mo, fi, gr	39.1	23.7	3.8	31.2	24.0	7.9	38.4	26.3	26.9
R	>15	1.4Y 6.5/0.8	Unweathered limestone									
**S2 (PDI** = **14.5)**												
Ah1	0–20	9.2YR 3.9/1.8	mo, me, gr	36.3	22.6	4.1	39.6	28.2	8.0	28.6	30.4	25.9
Ah2	20–32	9.2YR 5.1/2.1	mo, fi, sb	29.7	20.0	3.0	36.2	22.9	8.2	43.0	28.0	22.7
ACk	32–38	9.3YR 6.1/2.4	we, fi, sb	36.5	17.6	1.2	35.0	24.3	8.2	69.0	13.9	13.7
Ck	>38	1.8Y 5.9/0.9	Loose colluvial materials									
**S3 (PDI** = **18.0)**												
Ah1	0–11	9.9YR 2.9/2.0	mo, me, gr	33.1	39.7	2.7	46.3	28.7	7.7	27.8	49.0	31.8
Ah2	11–24	9.9YR 3.6/1.7	mo, me, sb	31.0	41.1	3.8	48.0	32.4	7.6	14.9	45.6	39.1
Bwk	24–42	8.3YR 2.7/1.8	st, me, sb	42.0	33.7	3.6	27.5	16.6	8.0	39.2	27.4	28.9
Ck	>42	1.0Y 6.9/0.6	Colluvial limestone fragments									
**S4 (PDI** = **19.9)**												
Ap	0–12	9.2YR 4.6/2.6	we, fi, gr	50.5	23.4	4.3	33.0	15.2	7.8	58.3	27.1	21.9
AB	12–40	8.9YR 4.5/2.7	we, me, sb	43.0	27.4	4.3	31.8	18.4	8.2	50.5	20.4	19.3
Bwk	40–58	8.3YR 5.5/3.4	mo, me, ab	35.2	31.0	5.1	35.2	17.6	8.2	61.4	13.5	14.9
Ck	>58	1.8Y 6.8/1.4	Colluvial limestone fragments									
**S5 (PDI** = **23.2)**												
O	0–4											
Ah1	4–14	8.3YR 4.3/2.9	mo, me, gr	29.3	33.1	3.4	34.5	24.8	7.5	43.6	53.0	24.2
Ah2	14–28	8.5YR 4.8/3.0	st, co, sb	27.2	38.5	3.8	37.4	26.4	7.9	47.0	34.9	25.0
AB	28–52	7.2YR 5.1/3.7	st, me, sb	23.3	37.5	4.1	35.2	20.0	8.1	45.0	21.0	18.8
Bwk	52–85	6.8YR 5.3/4.3	mo, me, ab	24.2	41.0	6.6	32.5	18.0	8.0	53.2	10.4	15.9
Ck	>85	0.1Y 6.0/1.6	Colluvial limestone fragments									
**S6 (PDI** = **24.7)**												
O	0–10											
Ah	10–32	8.2YR 3.2/1.7	st, me, gr	34.2	24.8	5.0	30.1	26.2	8.1	54.0	57.9	30.5
Bwk	32–65	8.0YR 5.4/3.3	st, me, sb	39.0	21.8	3.7	24.3	12.0	8.4	86.8	19.4	20.6
BCk	65–83	9.5YR 5.8/2.8	we, fi, sb	43.8	15.9	1.6	24.0	13.0	8.5	88.0	16.5	15.9
Ck	>83	2.0Y 6.0/2.2	Loose colluvial materials									
**S7 (PDI** = **25.2)**												
Ah	0–10	6.5YR 3.5/3.5	st, me, gr	7.5	53.1	24.4	39.0	23.5	7.6	0.0	58.9	38.0
AB	10–18	5.9YR 3.5/3.7	st, co, ab	7.3	60.8	25.3	33.0	25.4	7.8	0.0	37.5	32.1
Bt	18–45	5.0YR 3.5/3.8	st, co, ab	5.7	67.6	32.7	35.0	27.0	7.8	3.7	28.0	35.0
R	>45	2.5Y 6.0/0.9	Unweathered limestone									
**S8 (PDI** = **25.7)**												
Ap	0–18	7.0YR 4.0/3.4	st, co, ab	31.2	40.5	11.6	27.6	18.5	8.5	14.0	16.7	22.2
Btk	18–60	6.3YR 3.8/3.0	st, co, ab	30.7	45.0	18.6	25.9	18.0	8.3	19.6	12.0	26.5
Ck	>60	2.0Y 6.3/2.0	Loose colluvial materials									

^a^Grade: we: weak, mo: moderate, st: strong; Class: fi: fine, me: medium, co: coarse; Type: gr: granular. sb: subangular blocky, ab: angular blocky.

^b^Dithionite-extractable Fe.

^c^PDI: Harden’s profile development index.

The soils S3, S4, S5, and S6 exhibited a Bw horizon. The formation of a Ah surface organic horizon was also an outstanding pedogenic feature in S3 and S6, two Calcic Chernozems located on north-facing slopes at about 1500 m a.s.l., and in S5, a Calcic Kastanozem in a south-west-facing slope at 990 m a.s.l. The profile development indices in S5 and S6 were akin to the soils having a Bt horizon, because the soil thickness (83–85 cm, Table 1) offsets the lesser change in the Bw characteristics with respect to the parent material. However, their lower redness and contents of clay and Fe_d_ reflected a lower degree of development than in soils with a Bt horizon.

Finally, the soils S1 (Rendzic Leptosol) and S2 (Calcic Kastanozem) had horizon sequences of less pedogenic development (Ah-R and Ah-AC-C) and the lowest profile development indices (14.5 and 4.9), in accord with a microclimate, vegetation, and topography more restrictive to soil formation. Their pedoenvironments were south-facing slopes with sparse scrubs, resulting in soils of little thickness, brown colours and low contents in clay, Fe_d_, and organic C. The gain of organic matter in the A horizon was the only noticeable feature in the genesis of these soils, whereas the availability of water and erosion were the main constraints on pedogenesis. Thus, the soils ordered by their increasing profile-development index in Table 1 from S1 to S8 showed the studied pedogenic gradient. Consistent with an increase in weathering on the course of pedogenesis, the variation in clay and Fe_d_ contents of the subsurface horizon (AC or B) also allowed this gradient to be visualized and statistically represented (Supplementary Fig. S1a,b). However, the soil-profile development in S6 was due mainly to the accumulation of organic matter more than to weathering.

Table 2 lists the physicochemical indicators of soil quality determined in topsoil samples. Soil moisture, macroporosity, cation-exchange capacity, N, and K were positively correlated with organic carbon (organic C) content (*r* = 0.49 to 0.79, *P* < 0.05, *n* = 32). Soil moisture, available water, cation-exchange capacity, N, P, and K were also positively correlated with water-stable aggregates (*r* = 0.40 to 0.80). The pH and microporosity both correlated inversely with organic C and water-stable aggregates (*r* = −0.45 to −0.78). Consequently, the two latter parameters, also positively correlated with each other (*r* = 0.63, *P* < 0.0001, *n* = 32), these being the most informative of the soil-quality conditions. The amount of water-stable aggregates (mass of particles less than 250 µm in size forming stable aggregates) progressively increased in the soil sequence S1, S2, S4, S3, S5, S6, S7, and S8, and the organic C in a similar sequence although truncated in S4 and S8 (Supplementary Fig. S1c,d), suggesting that soil quality improved with increasing soil development but declined in organic C by the tillage.

**Table 2:** Soil quality along the pedogenic gradient of Sierra de María. Parameters measured (mean and standard deviation, *n* = 4) at the topsoil (20 cm).

Soil	Soil moisture (%)	Water-stable aggregates (Mg ha^−1^)	Macro porosity (cm^3^ cm^−3^)	Micro porosity (cm^3^ cm^−3^)	Available water (mm)	pH (H_2_O)	Organic C (Mg ha^−1^)	CEC (cmol_+_ kg^−1^)	N (Mg ha^−1^)	P (kg ha^−1^)	K (kg ha^−1^)
S1	5.3 (0.4)	370.3 a (54.2)	0.12 a (0.02)	0.35 b (0.03)	8.9 a (1.6)	8.3 c (0.2)	22.1 a (7.7)	20.5 ab (3.0)	1.2 (0.2)	82.9 (14.8)	77.3 (15.2)
S2	9.9 (1.8)	374.8 a (38.0)	0.40 d (0.04)	0.31 a (0.03)	7.1 a (1.1)	8.2 bc (0.1)	26.5 a (8.3)	26.3 c (3.2)	1.6 (0.3)	101.3 (41.0)	57.2 (9.3)
S3	19.0 (1.1)	489.7 a (99.1)	0.35 c (0.03)	0.31 a (0.01)	9.0 a (0.9)	7.7 a (0.2)	34.4 b (3.4)	31.5 d (2.8)	1.9 (0.3)	140.5 (41.2)	218.9 (65.1)
S4	5.7 (0.3)	481.1 a (64.2)	0.13 a (0.02)	0.40 c (0.03)	14.5 b (3.2)	8.1 bc (0.2)	19.4 a (3.2)	19.0 a (1.8)	1.4 (0.2)	121.7 (12.7)	104.3 (21.3)
S5	15.5 (1.8)	744.2 b (171.0)	0.34 c (0.04)	0.29 a (0.02)	15.9 b (2.9)	7.8 a (0.2)	49.8 c (3.2)	24.0 bc (3.4)	3.0 (0.7)	19.5 (3.3)	246.6 (45.3)
S6	24.0 (1.4)	814.8 bc (92.8)	0.27 b (0.02)	0.29 a (0.03)	7.4 a (2.1)	8.0 b (0.1)	47.7 c (5.1)	24.9 c (2.8)	1.7 (0.2)	62.8 (16.3)	195.0 (26.5)
S7	15.0 (1.1)	920.4 c (61.1)	0.32 c (0.02)	0.29 a (0.02)	15.1 b (1.5)	7.7 a (0.1)	44.7 c (4.0)	34.4 d (3.8)	3.2 (0.3)	245.8 (34.7)	400.2 (33.7)
S8	5.4 (0.5)	923.1 c (118.7)	0.15 a (0.03)	0.35 b (0.04)	16.6 b (2.8)	8.2 c (0.2)	22.7 a (3.3)	24.8 c (2.2)	1.6 (0.1)	404.2 (55.4)	299.8 (60.6)
*F-*ratio		24.2	70.8	14.7	14.5	13.5	23.3	13.5			
*H*	29.2								25.8	27.6	28.3

*F*-ratio is the statistic of ANOVA test within a column, in which mean values followed by different letters are significantly different (*P* < 0.05). When the assumptions for ANOVA were not met, the Kruskal-Wallis *H* test within a column indicated significant differences (*P* < 0.05) between medians.

### Composition and structure of the soil bacterial communities

Sequencing of 16S rRNA gene (V4-V5) amplicons with Illumina MiSeq and paired-read merging resulted in a total of 1,696,973 raw sequences. After eliminating the nonaligned ones and chimeras, we had a final number of 951,483 sequences (Supplementary Table S2) with an average length of 408 bp. High-quality reads from each soil were subsampled to 13,386 sequences (the lowest number obtained in sample S6B) prior to calculating alpha-diversity parameters. Rarefaction curves (Supplementary Fig. S2) did not show apparent saturation at the current surveying effort, which covered between 86% and 91% of the within-community (alpha) diversity (Supplementary Table S2). The indices Chao1, Inverse Simpson, Shannon, and Pielou tended to be lower in managed soils (S4 and S8) than in natural soils, but they were not related to the pedogenic gradient.

**Figure 2:**
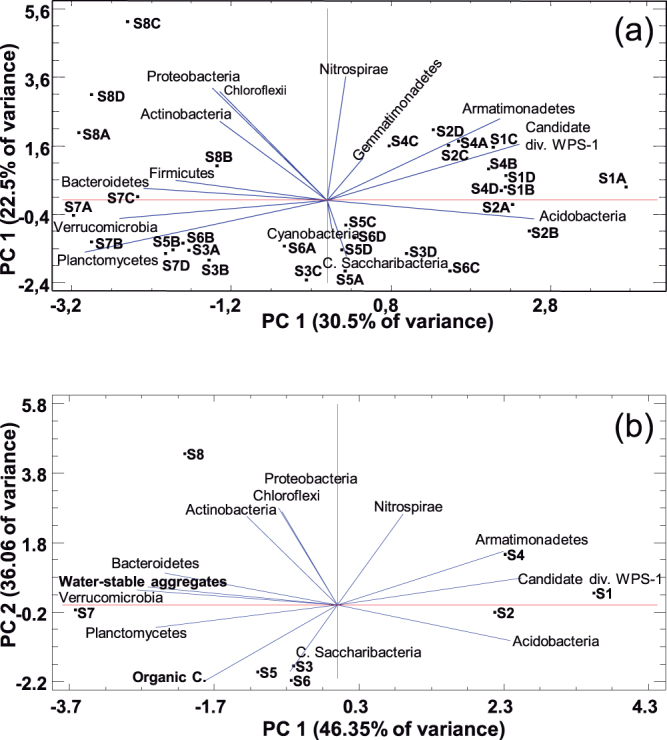
Principal component analysis. Biplots for the relative abundance of bacterial phyla (**a**), and selected bacterial phyla, organic C, and water-stable aggregates (**b**). The black markers are the soil scores S1 to S8 (four spatial replicates A, B, C, and D in (**a**), and mean values in (**b**)) and the blue vectors represent the loadings of variables.

The RDP classifier identified 14 phyla, with Acidobacteria, Bacteroidetes, Proteobacteria, Actinobacteria, and Candidate division WPS-1 being the most abundant (Table 3). A principal component analysis accounted for 74.1% of the bacterial variation at the phylum level in a solution of four principal components (Supplementary Table S3). The phyla (variables) with the longest positive loadings on PC1 (Acidobacteria, Canditate division WPS-1, and Armatimonadetes) influenced the scores mostly of soils S1, S2, and S4 with their spatial variability (replicates A, B, C, and D in Fig. 2a), whereas the variables with negative loadings (Bacteroidetes, Proteobacteria, Actinobacteria, Planctomycetes, Verrucomicrobia, etc.) influenced mainly the scores of S7 and S8. The alignment of soil scores along PC1 in Fig. 2a indicated changes in the content of the above phyla across the pedogenic gradient, from S1, S2, and S4 (richer in the phyla represented on the right), to S7 and S8 (richer in the phyla represented on the left), going through S3, S5, and S6 (in the middle, with intermediate contents). The progressive increase in the phyla represented on the right led to a decrease in those on the left and vice versa (Table 3). Likewise, the phyla with loadings of opposite signs (inverse correlation) in PC2 (Fig. 2a) showed differences between the soil group, S1, S2, S4, and S8, and the group S3, S5, S6, and S7. Two statistical comparison tests confirmed significant differences in the bacterial community structure of soils at the phylum level (Table 3).

**Table 3:** Abundance of bacterial phyla in the soils S1 to S8 (mean percentage and standard deviation, *n* = 4).

Bacteria	S1	S2	S3	S4	S5	S6	S7	S8	*F*-ratio	*H*
Acidobacteria	23.85 c (1.29)	26.21 d (1.28)	21.08 b (1.65)	21.57 b (1.63)	21.65 b (2.31)	20.85 b (2.71)	18.47 a (1.04)	17.85 a (1.71)	7.8	
Bacteroidetes	8.32 ab (1.18)	8.51 ab (0.97)	8.70 ab (0.19)	8.35 ab (0.58)	9.46 bc (1.10)	8.16 a (0.98)	11.05 d (0.77)	10.08 cd (0.78)	5.6	
Proteobacteria	5.46 a (0.57)	5.47 a (0.76)	5.09 a (0.51)	5.65 a (0.62)	4.81 a (0.65)	5.22 a (0.34)	5.80 a (0.73)	7.45 b (1.23)	5.0	
Actinobacteria	2.35 (0.59)	2.05 (0.25)	2.19 (0.13)	3.10 (0.28)	2.35 (0.92)	2.33 (0.44)	3.06 (0.20)	3.84 (1.08)		13.9
Candidate div. WPS-1	4.09 d (0.85)	2.83 c (0.10)	2.13 ab (0.17)	3.93 d (0.40)	2.74 bc (0.53)	2.45 bc (0.44)	1.78 a (0.13)	2.55 bc (0.25)	14.1	
Planctomycetes	1.32 a (0.20)	2.58 ab (2.15)	2.77 b (0.50)	1.34 a (0.39)	2.72 b (0.25)	2.39 ab (0.64)	3.55 c (0.23)	2.62 b (0.57)	15.6	
Verrucomicrobia	1.50 (0.12)	1.67 (0.46)	2.24 (0.56)	1.94 (0.59)	2.23 (0.39)	1.97 (0.25)	2.61 (0.74)	2.43 (0.27)		18.2
Gemmatimonadetes	1.55 (0.21)	2.69 (0.81)	1.32 (0.18)	1.67 (0.34)	2.13 (1.00)	0.85 (0.42)	1.95 (0.48)	1.54 (0.11)		19.0
Armatimonadetes	1.07 c (0.25)	0.73 b (0.05)	0.38 a (0.17)	1.00 c (0.25)	0.47 a (0.05)	0.52 ab (0.06)	0.47 a (0.13)	0.70 b (0.03)	11.8	
Others	5.20 (0.97)	7.86 (4.08)	7.15 (1.58)	5.94 (1.72)	7.88 (1.90)	6.13 (1.85)	8.85 (1.76)	8.22 (1.45)		
Not Classified	49.66 (1.19)	46.34 (2.73)	53.28 (0.68)	50.46 (1.51)	50.64 (4.13)	54.34 (1.83)	50.52 (1.11)	49.31 (1.86)		

*F*-ratio is the statistic of ANOVA test within a row, in which mean values followed by different letters are significantly different (*P* < 0.05). When the assumptions for ANOVA were not met, the Kruskal-Wallis *H* test within a row indicated significant differences (*P < *0.05) between medians.

A total of 139 taxa with relative abundance higher than 0.05% were identified at the lowest classification level (subgroup to genus). A principal component structure of fan-shaped vectors (variables) pointing to different soil scores (Supplementary Fig. S3) showed changes in the bacterial communities of soils. Acidobacteria Gp-4 and Armatimonadetes_gp4 were especially abundant in S1, S2, and S4; *Nocardioides* and *Terrimonas* progressively increased from S3 to S5, S6, and S7; whereas *Solirubrobacter* and *Microvirga* prevailed in S8. The statistical testing comparing soils revealed homoscedasticity (Levene’s test > 1.33, *P* > 0.265) in the first three variables with significantly different means (Supplementary Fig. S4), whereas the last three displayed significant differences in the medians (Kruskal-Wallis *H* test > 20.55, P < 0.004). Closer vectors in Supplementary Fig. S3 also identified other bacterial taxa strongly and positively correlated in abundance with each one of the above and, therefore, with a tendency to appear together in the same soil community as bacterial consortia (Supplementary Table S4).

### Relationships between bacteria and soil-quality parameters

Some bacteria such as Cyanobacteria, Firmicutes, and Gemmatimonadetes hardly correlated with the soil-quality parameters. However, a number of correlation coefficients in Candidate division WPS-1 and Armatimonadetes (Table 4) indicated that they tend to be more abundant with decreasing soil moisture, water-stable aggregates, macroporosity (increased microporosity), organic C, CEC, N, and K. In other words, these bacteria predominated in the soils of lower quality, also having the most alkaline pH. Just the opposite soil conditions were related to Planctomycetes. Acidobacteria and Actinobacteria also showed opposite behaviour (coefficients of the opposite sign) with respect to the amount of water-stable aggregates, P, and K. This was consistent with the strong inverse correlation between Acidobacteria and Actinobacteria (*r* = −0.82, *P* < 0.0001, *n* = 32).

**Table 4:** Pearson’s correlation coefficients (*P* < 0.05, *n* = 32) between soil-quality parameters^a^ and abundance of bacteria representative of the different soil communities.

	Moisture	WSA	Mpores	mpores	A.W.	pH	O.C.	CEC	N	P	K
Acidobacteria		−0.66			−0.50					−0.52	−0.58
Bacteroidetes		0.53			0.49	−0.37		0.45	0.55	0.54	0.69
Proteobacteria	−0.38	0.45								0.65	
Actinobacteria		0.54								0.38	0.41
Candidate div. WPS-1	−0.60	−0.57	−0.62	0.68		0.56	−0.57	−0.77	−0.57		−0.67
Planctomycetes	0.49	0.70	0.41	−0.56	0.39	−0.69	0.59	0.70	0.66	0.37	0.77
Verrucomicrobia	0.57				0.39	−0.41		0.40	0.40		0.60
Armatimonadetes	−0.71	−0.49	−0.64	0.70		0.72	−0.74	−0.61	−0.59		−0.53
Nitrospirae	−0.56					0.52	−0.49		−0.44		
Chloroflexi	−0.44				0.37					0.66	
C. Saccharibacteria	0.49						0.38				
Acidobacteria Gp4	−0.42	−0.60		0.36		0.49	−0.47	−0.48	−0.38		−0.56
Armatimonadetes_gp4	−0.62	−0.71	−0.46	0.64		0.71	−0.70	−0.62	−0.58		−0.67
*Terrimonas*	0.55	0.62	0.37	−0.53	0.36	−0.70	0.70	0.59	0.79		0.69
*Nocardioides*	0.63	0.52	0.38	−0.56		−0.69	0.62	0.59	0.56		0.56
*Solirubrobacter*		0.40								0.51	
*Microvirga*	−0.52		−0.41	0.40		0.50	−0.47			0.59	

^a^Moisture: soil mass wetness (%); WSA: Water-stable aggregates (Mg ha^−1^); Mpores and mpores: Macroporosity and microporosity (cm^3^ cm^−3^); A.W.: Available water (mm); pH; O.C.: Organic C (Mg ha^−1^); Cation exchange capacity (cmol_+_ kg^−1^); N: Total N (Mg ha-1); P and K: available P and K (kg ha-^1^).

Integrating these correlations into a principal component analysis with bacterial phyla representative of the different communities and the two most informative soil-quality parameters (water-stable aggregates and organic C), the PC1 showed that the decrease in Armatimonadetes, Candidate division WPS-1, and Acidobacteria from S1, S2, and S4 to the remaining soils, and the consequent relative increase in other phyla such as Proteobacteria, Actinobacteria, and Bacteroidetes, proved to be related mainly to the increase in water-stable aggregates (Fig. 2b). This discrimination of soil bacterial communities was also related to the organic C content, which had a certain loading on PC1. On the contrary, the separation of soil bacterial communities along the PC2 appeared to be related only to the organic C content, which was greater in S7 than S8 and also greater in S2 than S4.

**Figure 3:**
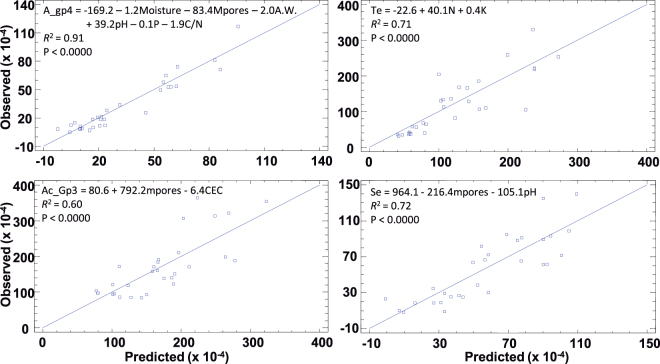
Multiple linear-regression models. Observed abundance (%) of Armatimonadetes_gp4 (A_gp4), *Terrimonas* (Te), Acidobacteria Gp3 (Ac_Gp3), and *Segetibacter* (Se) *versus* predicted abundance from physicochemical soil-quality parameters (Table 2). The entry of variables into the models by forward stepwise analysis was controlled by an *F*-ratio criterion of 4.

Several taxa of lower taxonomic categories also correlated with soil-quality parameters (Table 4), which may be indicating specific soil requirements for certain bacterial taxa. Thus, Acidobacteria Gp4 and Armatimonadetes_gp4, being representative of the bacterial consortia in S1, S2, and S4, were correlated to lower contents of water-stable aggregates and organic C, as well as unfavourable conditions of other soil parameters co-varying with them. Although *Solirubrobacter*, *Microvirga*, *Nocardioides*, and *Terrimonas* flourished mainly in soils having more stable aggregates, the two latter genera were also correlated with a lower pH and higher organic C content. Some relationships can be fit to regression models (Fig. 3), indicating that the influence of soil-quality parameters on the composition of bacterial populations is highly significant. Usually, the abundance of each taxon depended on several soil variables (multiple regressions) and not a single parameter. Like representative taxa of the different soil bacteria populations (Armatimonadetes_gp4 and *Terrimonas*), other consortiated taxa (Acidobacteria Gp3 and *Segetibacter*, Supplementary Table S4) also varied in abundance depending on soil-quality status.

## Discussion

The statistical analysis of the sequencing data showed variations in the distribution patterns of bacteria across the soils. The soils S1 and S2 were found to be richer in Acidobacteria, Candidate division WPS-1, and Armatimonadetes, while soils S7 and S8 had a significantly greater content in Actinobacteria, Proteobacteria, and Bacteroidetes (Fig. 2a, Table 3). Between these two soil groups, another with S3, S5, and S6 showed an intermediate bacterial composition. The soil S4 had high abundance in both Acidobacteria and Actinobacteria. Therefore, although it is postulated that microorganisms are globally dispersed and the soil environment is constantly invaded by unspecialized taxa, which having a high functional redundancy^30^ are randomly selected or acquiring the necessary functionalities by horizontal gene transfer^29,31,32^, the different bacterial community structures in our soils ordered along a pedogenic gradient could hardly be attributed exclusively to random.

The acidobacterial population decreased with increasing contents of water-stable aggregates, organic C, soil moisture, and nutrients (Table 4, Fig. 2b), i.e. when the soil-quality conditions improved. Unlike the work by Liu *et al*.^25^ in Mollisols, the abundance of Acidobacteria in our Mediterranean soils did not increase with organic C content. However, in agreement with these authors, different phylotypes were present in soils with different pH values (inversely related to organic C content). In particular, the changes in the bacterial consortia from Gp3 and Gp4 to Gp5, Gp10, and Gp18 (Supplementary Table S4) occurred as the soil became less alkaline and its content of organic C increased. With an improvement in soil quality, Actinobacteria, Proteobacteria, and Bacteroidetes flourished, the former being especially well established in the red soils S7 and S8, as well as being richer in water-stable aggregates cemented by organic C and/or secondary Fe oxides. All the above, together with the relations of Acidobacteria Gp4, Armatimonadetes_gp4, *Solirubrobacter*, *Nocardioides*, *Terrimonas*, and *Microvirga* with physicochemical parameters (Table 4) indicates that the soil bacterial community parallels the soil quality status. The abundance of certain bacteria depends on a combination of physicochemical parameters rather than a single parameter (Fig. 3), which means that it is influenced by the soil as a whole.

Two major latent factors, statistically referred to as dimensions or components, accounted for between 50% and 80% of the total bacterial variability in the eight soils (Fig. 2 and Supplementary Fig. S3). According to the distribution of soil scores, the first factor at the phylum level separated the poorly developed soils (S1 and S2) from the well-developed (S7 and S8), and between them the remaining soils of intermediate development (S3, S5, and S6), as reflected by their morphological and analytical characteristics including the profile development indices (Table 1). Thus, soil development could be a major driver in shaping the soil bacterial communities. Changes in the metagenomic abundance of bacteria along the studied pedogenic gradient can be explained pedologically in two senses. Firstly, this is because the soil is the result of the environmental factors: parent material, vegetation, topography, and (micro)climate^33^, and, secondly, because the development of soil changes the physicochemical parameters of inherent soil quality^34^. Environmental and physicochemical influences on the bacterial diversity have been reported from the earliest^9,14^ to the most recent studies^13,24^, but never before has the integrative influence of both been shown through soil development. In addition, this interpretation is consistent with the hypothesis that a large part of the genetic divergence of microbial assemblages can result from environmental factors^29^. Soil genesis summarizes these factors.

Bacterial communities linked to the most developed soils (communities represented by *Terrimonas*, *Nocardioides*, *Solirubrobacter*, and *Microvirga* in the Supplementary Table S4) also had a greater number of bacterial taxa positively correlated to each other than in the poorly developed soil profiles (represented by Acidobacteria Gp4 and Armatimonadetes_gp4). This indicates a greater interrelation within the bacterial assemblages of well-developed soils, suggesting that it is not due to chance, but presumably the result of their stabilization and adaptation through time. In addition, the preferential accumulation of *Solirubrobacter* and its consortium *Aciditerrimonas* in the most developed soils (Supplementary Table S4), which have been previously found, respectively, in reddish Saharan dust intrusions reaching Europe^35^ and linked to ferrous-ferric redox reactions^36^, may be connected with the partially aeolian origin proposed for red Mediterranean soils and their typical rubefaction process^37^. In this way, well-developed soils may help us to recognize the lingering effects of past evolutionary and ecological events on bacterial diversity.

A second latent factor (PC2 in Fig. 2) that at the phylum level separated the bacterial communities of forest soils S3, S5, S6, and S7 from the rest S1, S2, S4, and S8 appeared to be related in part to land use and management. Tillage has been identified as a major cause of soil compaction, loss of organic matter, and re-carbonation^38,39^; in addition to disturbing microbial communities^12,26,40^. Tillage can therefore explain the separation of the soils S4 (reforestation) and S8 (agriculture) from the rest by the second factor, but not that of native soils S1 and S2. However, S1, S2, S4, and S8 shared a low organic C content, which may be the main reason for this factor (Fig. 2), regardless of whether the cause is natural (scant soil development) or anthropogenic (soil degradation). This organic factor was the first to distinguish soil bacterial communities at taxonomic levels lower than phylum (Supplementary Fig. S3), whereas exclusively pedogenic effects still linger in the soil communities as a second factor.

The soil relationships at the phylum level could be indicative of an apparent evolutionary DNA sequence stability in the bacteria from our soils because of intermittent periods of dominance^28^. The stages of soil formation and degradation could be interpreted as stressed situations for the bacteria, which must be adapted to new environmental and soil-quality conditions. This stability could promote the abundance of certain phylogenetic groups according to the pedogenic environment, although we cannot disregard the presence of any bacterial taxon in a system as diversified as the soil^41^.

In conclusion, we have shown differences in bacterial community structures along the studied pedogenic gradient related to soil development and quality. Consequently, soil bacteria change with pedogenesis, which in turn depends on environmental factors and time. Thus, soil is a window into the evolutionary and environmental history of the soil bacterial communities.

## Methods

### Setting

Sierra de María was part of a marine carbonate platform along the palaeomargin of Iberian Massif, subsequently affected by the Alpine folding, and today included among the eastern mountains of the External Zones of the Betic Cordilleras in the Mediterranean region. Across an elevational range from 800 m to 2045 m and a substrate of limestone, the landscape consists of forestlands with extensive natural tree masses (*Pinus halepensis* Mill., *Quercus ilex* L., *Pinus nigra* Arnold), areas of degraded shrubs (*Quercus coccifera* L., *Juniperus phoenicea* L., *Vella spinosa* Boiss), and some reforested pine stands (*Pinus halepensis* Mill.). In addition, there are extensive rock outcrops and rangelands with scrubs (e.g. *Stipa tenacissima* L., *Lygeum spartum* L., and *Rosmarinus officinalis* L.) besides some patches of croplands in the low parts of slopes and valleys with cereal, almond trees, and olive trees. The climate is Mediterranean, somewhat semi-arid, and with an elevational gradient of mean annual rainfall and temperature, ranging between 250 mm and 17 °C in the lowest point, and 600 mm and 6 °C at the highest peak.

### Soil sampling

Following previous soil surveys^42^, a field judgement sampling was performed in order to select sites of the climax pedoenvironments with well-developed soils such as Luvisols, Chernozems, and Kastanozems as well as sites of the young and poorly developed soil units with Calcisols and Leptosols. Finally, we selected eight sites within a maximum distance of about 20 km, including natural and managed soils. At each site a soil pit was dug to inspect the soil profile and carry out a discrete depth sampling by natural horizons. In addition, after removing the litter O layer, we collected composite topsoil samples in four plots of 3 × 3 m laid out in a cross pattern at a distance of 20 m from the soil profile (Fig. 1). Each consisted of a bulk soil material (250 cm^3^) of the upper 20 cm from each corner, from the midpoint of the sides, and from the centre of the square plot, which then were thoroughly mixed to make a composite topsoil sample. A subsample was transported to the laboratory in isothermal bags, sieved through a 4 mm screen, and stored in polythene containers at −80 °C for subsequent next-generation sequencing (NGS) analysis. The other topsoil subsample and each of the soil-horizon samples were air dried, crumbled, and sieved to 2 mm for subsequent physicochemical analyses. We also took intact cores in stainless-steel cylinders to measure soil bulk density and moisture with respect to dry soil weight at 105 °C. Samplings were taken in early June as the period of highest biological activity after the spring rains.

### Physicochemical analysis

Soil structure and consistency were determined according to FAO guidelines for soil description^43^ and the Munsell colour measured with a Konica Minolta CM-2600d spectrophotometer (Minolta Co. Tokyo). In fine earth (<2 mm) and following standard procedures^44,45^, we analysed the particle-size distribution by sieving (sand) and by the pipette method (silt and clay) after removing organic matter with H_2_O_2_ and dispersion with sodium hexametaphosphate. In addition, the soil-water release at −33 kPa and −1500 kPa was measured on a Richard’s membrane, particle density with a pycnometer, and water-stable aggregates using a wet sieving apparatus with a mesh size of 250 µm (Eijkelkamp Co., Giesbeek, The Netherlands). We also determined the pH by potentiometry in a 1:1 soil:water suspension, cation-exchange capacity and exchangeable bases using ammonium and sodium displacement solutions, as well as the contents of organic C by dichromate oxidation, total N by the Kjeldhal method, available P by ammonium acetate extraction followed by colorimetry, equivalent CaCO_3_ with a Bernard’s calcimeter, and dithionite-extractable Fe as described in Mehra and Jackson (1960)^46^.

Using the data compiled from the above analyses, we calculated the Harden’s profile development index^47^ and, on a fine-earth volume basis in the top 20 cm of soil^48,49^, the contents of available water, water-stable aggregates, organic C, total N, extractable P, exchangeable K, total porosity from the particle and bulk density, microporosity estimated as water volume at field capacity (−33 kPa), and macroporosity from total porosity less microporosity.

### DNA extraction and PCR amplification

Total genomic DNA was extracted from 0.25 g of each individual topsoil sample following the manufacturer’s protocol of the PowerSoil™ DNA Extraction Kit (MoBio Laboratories Inc., Carlsbad, CA, USA). DNA size and quality was checked by electrophoresis in 1.5% (w/v) agarose gel. Ten ng of total DNA were used as a template for the PCR amplification of the 16S rRNA gene hypervariable V4-V5 regions in the topsoil samples. PCR amplification conditions consisted in 25 cycles using 55 °C as annealing temperature as described by Aguirre-Garrido *et al*.^50^.

### Illumina-Sequencing of 16S V4-V5 amplicons

PCR amplification products of the V4-V5 variable regions of the 16S rRNA gene were obtained using fusion universal primers 515 F (Illumina adaptors + 5′GTGYCAGCMGCCGCGGTAA3′) and 926 R (Illumina adaptors + 5′CCGYCAATTYMTTTRAGTTT3′). Amplicon multiplexing and sequencing was carried out with a dual indexing tag-tailed design using 8nt indices from Nextera XT Index Kit v2 (Illumina, San Diego, CA, USA). Paired-end sequencing of 16S PCR amplicon libraries was performed using the Illumina MiSeq instrument with 300 + 300 v3 kit chemistry at Centre for Comparative Genomics and Evolutionary Bioinformatics (CGEB)-Dalhousie University, Canada.

### Bioinformatic analysis

The 16S rRNA data were processed with MOTHUR software v. 1.39.5^51^. following the MiSeq SOP^52^. Once demultiplexing with a 1 bp mismatch in the barcodes and 2 pb mismatches in the primer, chimeric reads were identified and excluded using Chimera UCHIME^53^. Diversity was examined by operational taxonomic units (OTUs) considering quality reads at 3% dissimilarity and the distance-based greedy clustering algorithm (dgc), as well as rarefaction curves computed at 97% similarity with the Mothur’s alpha diversity pipeline. The number of observed OTUs and the indices Chao1 richness, Inverse Simpson diversity, Shannon diversity, and Pielou evenness were finally calculated by the equations described and implemented in Mothur. Finally, we determined the composition of bacterial communities with the RDP Bayesian classifier Trainset 14, fixrank classification^54^. Only the sequences that could be classified at the lower classification level (subgroup to genus) were used for further analysis. Abundance was expressed as a percentage with respect to the total number of sequences in each sample. Taxa with relative abundance higher than 0.05% were retained for statistical analysis.

### Statistical analysis

Principal component analysis performed with Statgraphic Centurion XVI (Statpoint Technologies, Inc., Warrenton), was applied to the data in order to reduce a large amount of information to a small number of orthogonal dimensions in such a way that they account for as much variation of the data set as possible^55^. This analysis transforms a number of possibly correlated variables (e.g. bacteria taxa) into a limited number of uncorrelated variables called principal components, which are linear combinations of the original variables. The resulting equation with the loadings (coefficients) of variables in each component allows calculating a score per soil sample. In graphical terms, a variable is denoted by a vector, while a soil score is represented by a point. Correlation and regression analysis were also occasionally applied to the data. Finally, we compared the data of each of the eight soils (4 replicates) on each variable using one-way ANOVA. The *F*-ratio determined whether there were significant differences between means and the multiple-range test of Fisher’s least significant differences showed which means were significantly different from others. Alternatively, when the assumptions for ANOVA were not met (like the assumption of homoscedasticity), we used the Kruskal-Wallis test determining whether the medians differ according to the *H* statistic^56^.

### Data availability

Sequence data were deposited in the Sequence Read Archive (SRA) of the National Centre for Biotechnology Information (NCBI) under the bioproject number PRJNA414389.

## Electronic supplementary material

Supplementary Information
