# Supplementary material for: Changes in the soil bacterial community along a pedogenic gradient
Source: Sci Rep. 2017 Nov 6;7:14593. doi: 10.1038/s41598-017-15133-x (PMC5674076; doi:10.1038/s41598-017-15133-x)
Supplement: Supplementary file 1 — Supplementary Information [file 41598_2017_15133_MOESM1_ESM.pdf]

Changes in the soil bacterial community along a pedogenic gradient

Manuel Sánchez-Marañón[1], Isabel Miralles[2], José F. Aguirre-Garrido[3], Manuel Anguita-Maeso[2,4], Vicenta Millán-Casamayor[5], Raul Ortega[2], José A. García-Salcedo[4,6], Francisco Martínez-Abarca[5]* & Miguel Soriano[2,4]*.

[1]Department of Soil Science and Agricultural Chemistry, University of Granada, E-18071 Granada, Spain. [2]Center for Intensive Mediterranean Agrosystems and Agri-food Biotechnology (CIAMBITAL), University of Almeria, E-04001 Almería, Spain. [3]CBS Universidad Autónoma Metropolitana-Lerma, Hidalgo Pte. 46, Col. La Estación, 52006 Lerma, Estado de México, México. [4]Pfizer-University of Granada-Junta de Andalucía Centre for Genomics and Oncological Research (GENYO), E-18016 Granada, Spain. [5]Molecular Ecology Group, Department of Soil Microbiology and Symbiotic Systems, Estación Experimental del Zaidín, Spanish Council for Scientific Research (EEZ-CSIC), E-18008 Granada, Spain. [6]Infectious diseases and Microbiology Unit, Biosanitary Research Institute ibs.GRANADA, University Hospitals of Granada / University of Granada, E-18012, Granada, Spain.

SUPPLEMENTARY INFORMATION

Supplementary Figures S1–S4

Supplementary Tables S1–S4

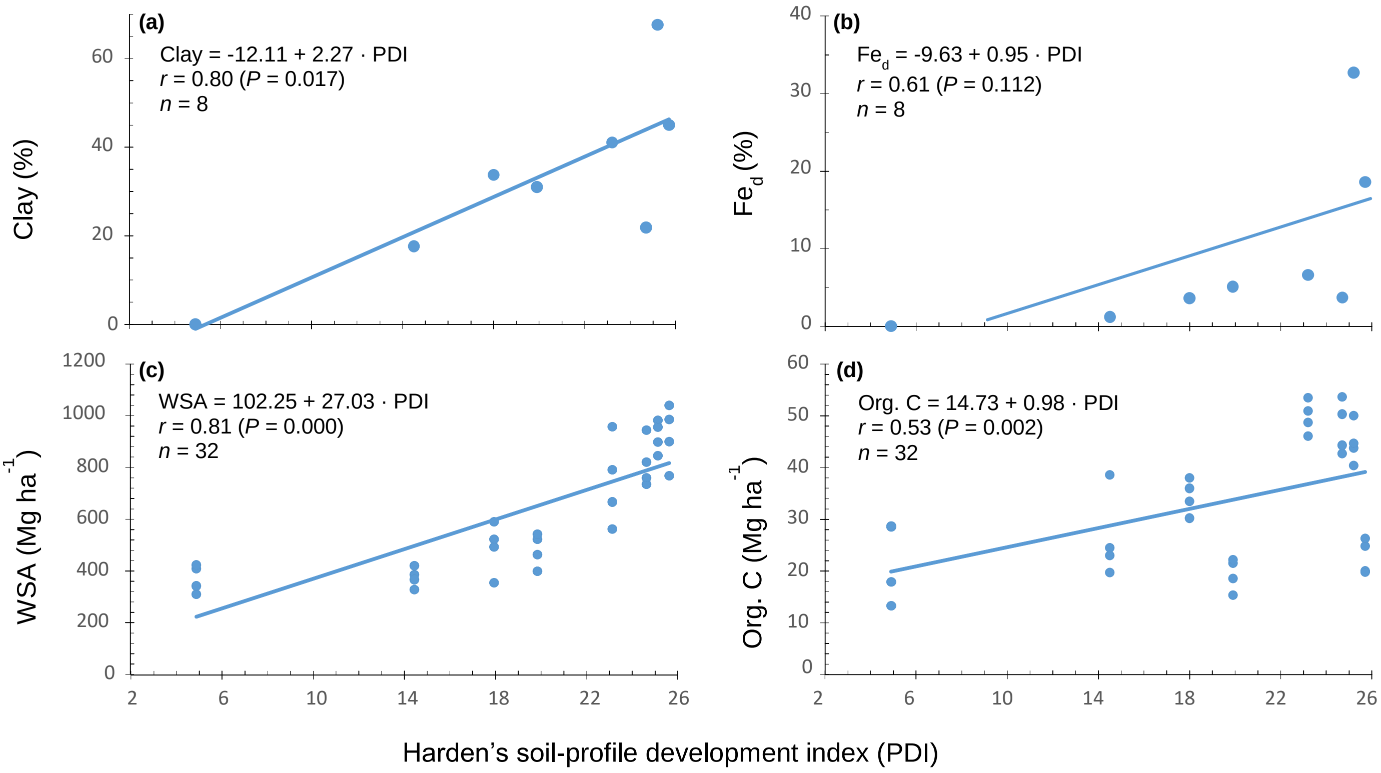

Supplementary Figure S1. Relationships between the soil-profile development index and the contents of clay and dithionite-extractable iron in the AC or B horizons of the soil profiles, as well as water-stable aggregates and organic C in the topsoil.

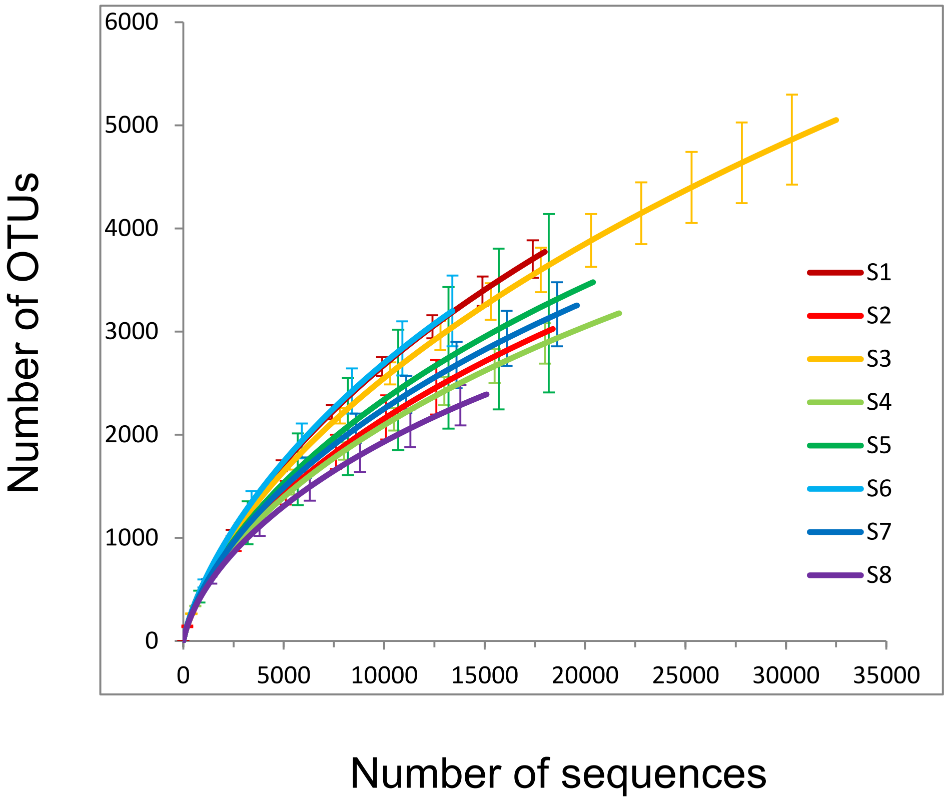

Supplementary Figure S2. Rarefaction curves for the topsoil samples (four replicates per site). Error bars represent the standard deviation.

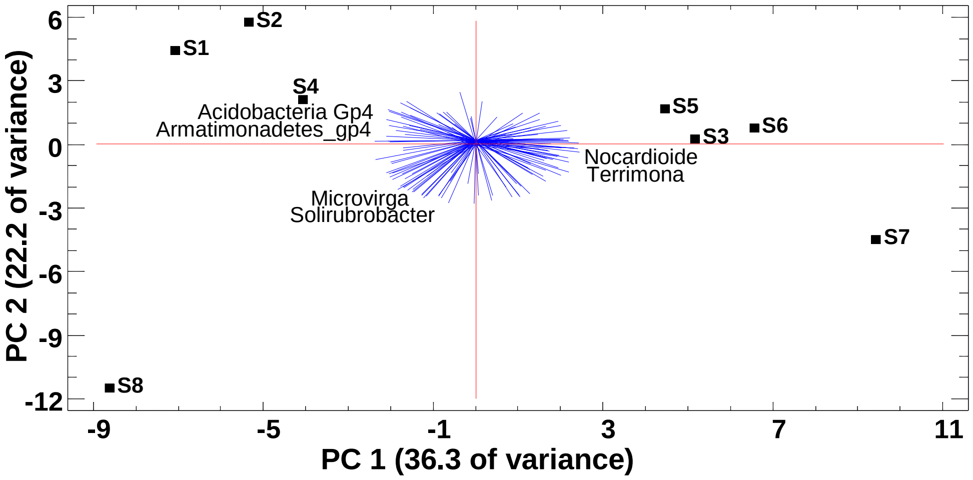

Supplementary Figure S3. Principal component analysis. Biplots for the abundance of bacterial taxa at the lowest classification level (subgroup to genus). The black markers are the soil scores S1 to S8 (mean values of four spatial replicates) and the blue vectors represent the loadings of variables (139 bacterial taxa).

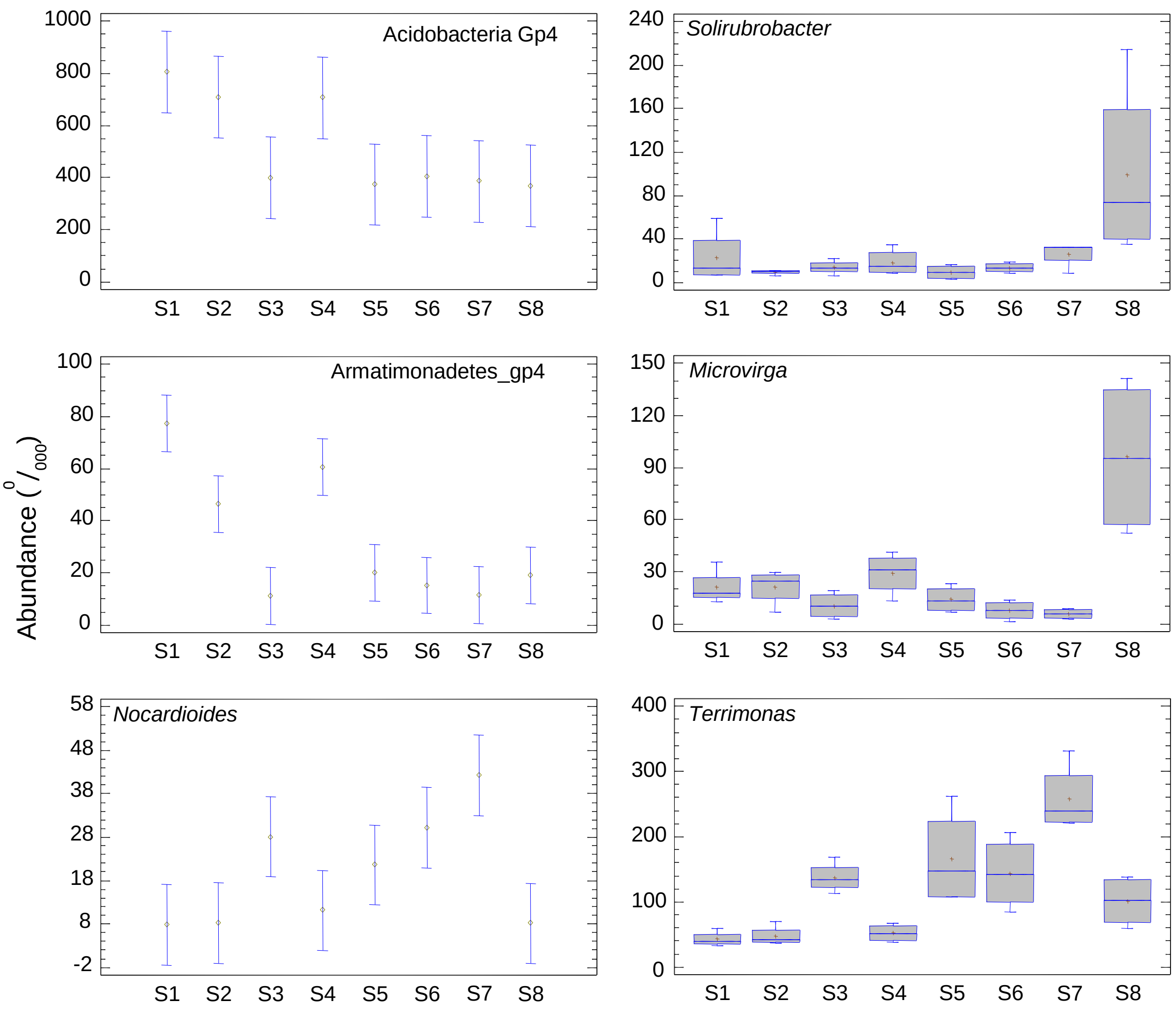

Supplementary Figure S4. Mean plots (± 95% of Fisher's LSD) of Acidobacteria Gp4, Armatimonadetes_gp4, and *Nocardioides,* as well as box and whisker plots of *Terrimonas, Microvirga*, and *Solirubrobacter* in the soils S1 to S8.

Supplementary Table S1. General characteristics of the sampling sites.

Soil id.	Soil typology	Geographic coordinates	Land Use/Vegetation	Altitude (m)	Orientation and slope
S1	Rendzic Leptosol	02º 08' 48" W, 37º 39' 26" N	*Stipa tenacissima* L. *Rosmarinus officinalis* L. and *Lygeum spartum* L. scrub	1140	SE-facing shoulderslope of 17%
S2	Calcic Kastanozem	02º 01' 38" W, 37º 48' 10" N	*Juniperus phoenicia* L. shrub	892	SW-facing backslope of 25%
S3	Calcic Chernozem	02º 12' 32" W, 37º 41' 31" N	*Quercus ilex* L. forest	1398	NW-facing backslope of 45%
S4	Haplic Calcisol	02º 05' 32" W, 37º 39' 58" N	Reforested *Pinus halepensis* Mill forest	1053	E-facing backslope of 16%
S5	Calcic Kastanozem	02º 02' 40" W, 37º 43' 50" N	*Pinus halepensis* Mill forest	990	SW-facing footslope of 38%
S6	Calcic Chernozem	02º 13' 08" W, 37º 40' 31" N	*Pinus nigra* Arnold forest	1520	N-facing backslope of 40%
S7	Leptic Luvisol	02º 00' 52" W, 37º 45' 06" N	*Quercus ilex* L. forest	1122	N-facing backslope of 33%
S8	Luvic Calcisol	02º 10' 22" W, 37º 42' 20" N	Almond tree crop	1230	N-facing footslope of 11%

Supplementary Table S2. Number of qualified reads and alpha diversity parameters. Good's coverage and number of OTUs; estimators of species richness (Chao 1), sample diversity (Inv-Simpson,H'), and evenness (J' Pielou) of the bacterial communities in soils S1 to S8 (Sierra de María, SE Spain).

Soil	Number of sequences[a]	Coverage[c]	OTUs[bc]	Chao 1[c]	Inv-Simpson[c]	H'[c]	J'[c]
S1	26205* (5684)	0.86 (0.02)	3187 (122)	6673.04 (1319.69)	255.96 (24.24)	6.78 (0.07)	0.71 (0.01)
S2	28642 (14105)	0.89 (0.02)	2546 (279)	5127.50 (1319.84)	156.06 (34.39)	6.34 (0.15)	0.67 (0.02)
S3	37876 (4138)	0.86 (0.01)	3042 (148)	6816.09 (1167.54)	212.86 (9.99)	6.65 (0.02)	0.70 (0.01)
S4	40208 (13185)	0.90 (0.01)	2463 (140)	4929.83 (914.68)	195.40 (13.12)	6.42 (0.04)	0.68 (0.01)
S5	25672 (4060)	0.88 (0.04)	2768 (690)	5425.87 (1794.50)	189.78 (46.03)	6.50 (0.35)	0.68 (0.04)
S6	31515 (15582)	0.86 (0.02)	3200 (300)	6440.77 (1380.56)	255.93 (58.47)	6.78 (0.18)	0.71 (0.02)
S7	36519 (11512)	0.89 (0.02)	2653 (220)	5261.74 (1137.17)	222.65 (7.28)	6.54 (0.07)	0.69 (0.01)
S8	27113 (12685)	0.91 (0.01)	2252 (194)	4194.70 (944.93)	194.38 (21.39)	6.34 (0.11)	0.67 (0.01)

[a] Number of quality sequences

[b] Number of observed operational taxonomic units (OTUs), defined on the basis of a 0.03 cutoff value.

[c] The smaller library (13,386 in sample S6B) was used for normalization of data.

[*]Mean and standard deviation (between brackets) of 4 replicates for each soil.

Supplementary Table S3. Component loadings on a four-component principal-component-analysis solution for the soil bacterial composition at the phylum level (n = 32).

Variable	PC1	PC2	PC3	PC4
Acidobacteria	0.376	-0.103	-0.346	0.195
Bacteroidetes	-0.336	0.053	-0.264	-0.268
Proteobacteria	-0.196	0.425	0.107	-0.087
Actinobacteria	-0.187	0.311	0.371	0.184
C. div. WPS-1	0.349	0.216	0.183	-0.210
Planctomycetes	-0.431	-0.196	-0.115	-0.086
Verrucomicrobia	-0.380	-0.068	0.043	-0.073
Gemmatimonadetes	0.069	0.149	-0.592	0.291
Armatimonadetes	0.309	0.319	0.095	-0.248
Nitrospirae	0.044	0.471	-0.186	0.300
Chloroflexi	-0.208	0.430	0.081	0.209
C. Saccharibacteria	0.019	-0.242	0.317	0.408
Firmicutes	-0.275	0.081	-0.230	0.236
Cyanobacteria	0.010	-0.147	0.241	0.537
Variance, %	30.521	22.439	11.997	9.110

Supplementary Table S4. Bacterial consortia. Taxa strongly ($P < 0.001$) and positively correlated in abundance with Acidobacteria Gp4, Armatimonadetes_gp4, *Terrimonas*, *Nocardioides*, *Solirubrobacter*, and *Microvirga* ($n = 32$).

Acidobacteria Gp4 $r = 0.57$-0.74	Armatimonadetes_gp4 $r = 0.61$-0.80	Terrimonas $r = 0.55$-0.84	Nocardioides $r = 0.56$-0.82	Solirubrobacter $r = 0.59$-0.97	Microvirga $r = 0.50$-0.92
	Acidobacteria Gp3	Acidobacteria Gp18	Acidobacteria Gp5		
	Acidobacteria Gp4		Acidobacteria Gp10		
			Solibacter		
Spirosoma	Dyadobacter	Segetibacter	Segetibacter	Chitinophaga	Chitinophaga
		Adhaeribacter	Terrimonas	Cnuella	Flavisolibacter
		Larkinella	Larkinella		
Geminicoccus	Geminicoccus	Altererythrobacter	Minicystis	Reyranella	Reyranella
Aquicella	Aquicella	Mesorhizobium	Povalibacter	Microvirga	Duganella
Sphingomonas	Sphingomonas	Burkholderia	Burkholderia	Pseudolabrys	Pseudolabrys
		Phyllobacterium	Phyllobacterium	Luteimonas	Luteimonas
		Hyphomicrobium	Hyphomicrobium	Rhodoferax	Rhodoferax
				Variibacter	Variibacter
				Rhizobacter	
		Pseudonocardia	Propionibacterium	Blastococcus	Blastococcus
		Nocardioides	Rubrobacter	Streptomyces	Streptomyces
		Arthrobacter	Arthrobacter	Aciditerrimonas	Aciditerrimonas
		Blastococcus	Agromyces	Asanoa	Asanoa
				Saccharothrix	Saccharothrix
					Solirubrobacter
		Schlesneria	Schlesneria	Planctopirus	Planctopirus
		Gemmata	Gemmata		Blastopirellula
			Telmatocola		
Armatimonadetes_gp4				Armatimonadetes_ gp5	Armatimonadetes_ gp5
		Kallotenue		Nitrolancea	Nitrolancea
		Opitutus			
			Staphylococcus		
					Nitrospira
